# Decreased Plasma IL-35 Levels Are Related to the Left Ventricular Ejection Fraction in Coronary Artery Diseases

**DOI:** 10.1371/journal.pone.0052490

**Published:** 2012-12-21

**Authors:** Yingzhong Lin, Ying Huang, Zhengde Lu, Cheng Luo, Ying shi, Qiutang Zeng, Yifeng Cao, Lin Liu, Xiaoyan Wang, Qingwei Ji

**Affiliations:** 1 Department of Cardiology, the People’s Hospital of Guangxi Zhuang Autonomous Region, Nanning, China; 2 Department of Ultrasound, The People’s Hospital of Guangxi Zhuang Autonomous Region, Nanning, China; 3 Institute of Cardiovascular Diseases, Union Hospital, Tongji Medical College, Huazhong University of Science and Technology, Wuhan, China; Heart Center Munich, Germany

## Abstract

**Background:**

Accumulating evidence shows that the novel anti-inflammatory cytokine IL-35 can efficiently suppress effector T cell activity and alter the progression of inflammatory and autoimmune diseases. The two subunits of IL-35, EBI3 and p35, are strongly expressed in human advanced plaque, suggesting a potential role of IL-35 in atherosclerosis and coronary artery disease (CAD). However, the plasma levels of IL-35 in patients with CAD have yet to be investigated.

**Methods:**

Plasma IL-35, IL-10, TGF-β1, IL-12 and IL-27 levels were measured using an ELISA in 43 stable angina pectoris (SAP) patients, 62 unstable angina pectoris (UAP) patients, 56 acute myocardial infarction (AMI) patients and 47 chest pain syndrome patients as a control group.

**Results:**

The results showed that plasma IL-35 levels were significantly decreased in the SAP group (90.74±34.22 pg/ml), the UAP group (72.20±26.63 pg/ml), and the AMI group (50.21±24.69 pg/ml) compared with chest pain syndrome group (115.06±32.27 pg/ml). Similar results were also demonstrated with IL-10 and TGF-β1. Plasma IL-12 and IL-27 levels were significantly increased in the UAP group (349.72±85.22 pg/ml, 101.75±51.42 pg/ml, respectively) and the AMI group (318.05±86.82 pg/ml, 148.88±68.45 pg/ml, respectively) compared with chest pain syndrome group (138.68±34.37 pg/ml, 63.60±22.75 pg/ml, respectively) and the SAP group (153.84±53.86 pg/ml, 70.84±38.77 pg/ml, respectively). Furthermore, lower IL-35 levels were moderately positively correlated with left ventricular ejection fraction (LVEF) in CAD patients (R = 0.416, *P*<0.01), whereas higher IL-27 levels were weakly negatively correlated with LVEF in CAD patients(R = −0.205, *P*<0.01).

**Conclusions:**

The results of the present study show that circulating IL-35 is a potentially novel biomarker for coronary artery disease. Regulating the expression of IL-35 also provides a new possible target for the treatment of atherosclerosis and CAD.

## Introduction

Coronary artery disease (CAD) is the leading cause of hospitalization and the predominant contributor to mortality in China. It is now widely spread accepted that atherosclerosis is not only merely a lipid disorder but also a chronic inflammatory disease [1]. Inflammatory cells and pro-inflammatory cytokines are found in both early lesions and advanced lesions, as well as following plaque rupture and during the thrombus formation stage. Anti-inflammatory cytokines, however, do not match the increase of pro-inflammatory cytokines; therefore, the imbalance of anti-inflammatory and pro-inflammatory cytokines results in the progression of atherosclerosis, plaque instability and the subsequent onset of acute coronary syndrome (ACS, including unstable angina pectoris and acute myocardial infarction) [2]–[4].

IL-10 and TGF-β1, critical anti-inflammatory cytokines, have been widely investigated in atherosclerosis. Increased evidence indicates that decreased IL-10 and TGF-β1 levels are accompanied by the onset of acute coronary syndrome and that high concentrations of IL-10 and TGF-β1 could improve the prognosis of patients with coronary artery disease [5]–[11]. IL-35, a novel IL-12 family member, was identified in 2007 [12], [13]. IL-35 is a heterodimer cytokine comprising the p35 subunit of IL-12 and the subunit Epstein-Barr virus (EBV) -induced gene 3 (EBI3) which was identified in B lymphocytes based on its induction following EBV infection [12]–[15]. IL-35 is constitutively secreted by CD4+ regulatory T (Treg) cells rather than CD4+ effector T (Teff) cells in mice; therefore, IL-35 has been considered to be the characteristic factor of Treg cells [12], [13], [16]–[19]. Human Treg cells, placental trophoblast cells, activated dendritic cells, and macrophages also express IL-35 [20]–[23]. Additional evidence demonstrated that IL-35 is an important anti-inflammatory cytokine and can efficiently suppress the Teff cell (including Th1, Th2 and Th17 ) activity and reduce the progression of inflammatory diseases and autoimmune diseases [12], [13], [22]–[25]. Specifically, EBI3 and p35 are strongly expressed in advanced atherosclerotic plaque in humans and can be up-regulated by TNF-α and IFN-γ stimulation in cultured human primary aortic smooth muscle cells, suggesting IL-35 may be involved in atherosclerosis progression [26].

However, current information on the levels of plasma IL-35 and its potential role in coronary artery disease is still limited. In the present study, we measured the levels of plasma IL-35, IL-10 and TGF-β1 in CAD patients and their relationship to the other parameters of CAD, including plasma lipoproteins, C-reactive protein (CRP), coronary stenosis and left ventricular ejection fraction. Because IL-12, IL-27 and IL-35 belong to the IL-12 family and IL-35 interacts with both IL-12 through p35 and IL-27 through EBI3, the levels of plasma IL-12 and IL-27 were also measured in this study.

## Study Subjects and Methods

### Study Subjects

We recruited 208 patients who underwent diagnostic coronary angiography between July 2011 and May 2012 in the People’s Hospital of Guangxi Zhuang Autonomous Region, China. The patients were classified into 4 groups:

*1*: Stable angina pectoris (SAP) group (31 men and 12 women, mean age 61.6±12.3). Inclusion criteria: typical exertion-induced chest discomfort associated with down-sloping or a horizontal ST-segment depression >1 mm in an exercise test;

*2*: Unstable angina pectoris (UAP) group (40 men and 22 women, mean age 63.8±10.2). Inclusion criteria: chest pain at rest with definite ischemic electrocardiographic changes: ST-segment changes and/or T-wave inversions;

*3*: Acute myocardial infarction (AMI) group (39 men and 17 women, mean age 64.3±11.0). Inclusion criteria: myocardial infarction confirmed by significant rise of troponin I and creatine kinase MB levels;

*4*: Chest pain syndrome (CPS) group (31 men and 16 women, mean age 61.4±8.9) whose chest pain was not accompanied by ECG changes, coronary stenosis, or coronary spasm when an intracoronary injection of acetylcholine was given during coronary angiography.

Patients with AMI were treated with aspirin enteric-coated tablets 300 mg plus clopidogrel 300 mg one time in the emergency unit, followed with aspirin enteric-coated tablets 100 mg plus clopidogrel 75 mg daily. Those patients with SAP and UAP were treated with aspirin enteric-coated tablets 100 mg plus clopidogrel 75 mg daily. Clopidogrel is used alone when aspirin is absolutely contraindicated. Other medications such as β-blockers and angiotensin-converting enzyme inhibitors (ACEI) were treated according to the clinical condition of the patients.

Written informed consent was obtained from each patient. The study was approved by the Ethics Committee of the People’s Hospital of Guangxi Zhuang Autonomous Region, Nanning, China. The permit number of this study is “[2011] GS018.” Patients with valvular heart disease, thromboembolism, collagen disease, disseminated intravascular coagulation, advanced liver disease, renal failure, malignant disease, septicemia or on steroid therapy were excluded from the study.

### Blood Sample Collection and Processing

In the AMI group, blood samples were obtained upon the arrival of patients to the emergency unit. Fasting blood samples were obtained the morning following admission for the remainder of the study groups. Samples were collected into Sodium Heparin Vacutainers (Becton- Dickinson) and processed within 1 hour of collection by centrifugation for 15 min at 1000×g. The supernatant was then aliquoted and stored at −80°C until analysis. All samples were thawed only once.

### ELISA Detection of the Levels of Plasma Cytokines

The levels of plasma IL-35, IL-10, TGF-β1, IL-12 and IL-27 were measured using an enzyme-linked immunosorbent assay (ELISA), according to the manufacturer’s instructions (Westtang Bio-tech, Shanghai, China). Intra-assay and inter-assay coefficients of variation for ELISA were <5% and <10%, respectively. All samples were measured in duplicate.

### Effects of Aspirin and Clopidogrel on the Secretion of Cytokines

To investigate whether IL-35 production is rapidly affected by oral aspirin and clopidogrel *in vivo*, 60 health college students (aged from 19 to 25 years) from Guangxi Medical University were randomized into three groups (each containing 20 cases), receiving aspirin 100 mg, clopidogrel 75 mg or aspirin 100 mg plus clopidogrel 75 mg for once, respectively. Blood samples were collected before treatment and 24 hours after treatment. Plasma cytokines concentrations were measured using an ELISA.

### Gensini Score

The severity of coronary stenosis in patients was estimated by the Gensini coronary score following coronary angiography. The Gensini score was computed by assigning a severity score to each coronary stenosis according to the degree of luminal narrowing and its geographic importance. Reduction in the lumen diameter and the roentgenographic appearance of concentric lesions and eccentric plaques were evaluated (reductions of 25, 50, 75, 90, and 99% and complete occlusion were assigned Gensini scores of 1, 2, 4, 8, 16, and 32, respectively).The score was then multiplied by a factor that incorporates the importance of the lesion’s position in the coronary arterial tree as follows: 5 for the left main coronary artery; 2.5 for the proximal left anterior descending coronary artery (LAD) or left circumflex artery (LCX), 1.5 for the mid-LAD; and 1 for the distal LAD, the right coronary artery or the mid-distal LCX.

### Left Ventricular Ejection Fraction assessment

We applied Biplane Simpson’s method to quantify the left ventricular ejection fraction (LVEF) in 2-dimensional echocardiography. A GE ViVid E7 ultrasonography machine (GE Healthcare, America) with a transthoracic 1.5–4.3 MHz probe (M5S-D) was used for 2-dimensional echocardiography. Two-dimensional echocardiographic examinations were performed on the subjects in the left lateral decubitus position. Standard 4-chamber apical view and 2-chamber view were performed, and the dynamic 2-dimensional gray images were obtained at 60–100 frames per second.

### Statistical Analysis

All data are given as the mean±SD. The data were analyzed by an ANOVA. Where significance was found, a Newman–Keuls test was performed for post-hoc analysis to detect the difference among groups. Spearman’s correlation was used to calculate correlations between plasma cytokines levels and the other measured parameters. In all tests, a value of P<0.05 was considered to be statistically significant.

## Results

### 1. Basic Clinical Characteristics of Patients

There were no significant differences in age, gender, history of hypertension, diabetes or tobacco use among the four groups. The Gensini score and plasma CRP levels were significantly higher in CAD patients than in the chest pain syndrome group. The LVEF was lower in the SAP and AMI groups than in the chest pain syndrome group. The other parameters of each group, including lipid and lipoprotein fractions, fasting glucose, and pre-hospital medications are listed in Table 1.

**Table 1:** Clinical characteristics of patients.

Characteristics	CPS	SAP	UAP	AMI
	(n = 47)	(n = 43)	(n = 62)	(n = 56)
Age (years)	61.4±8.9	61.6±12.3	63.8±10.2	64.3±11.0
Sex (male/female)	31/16	31/12	40/22	39/17
Hypertension, n(%)	28 (59.6)	24 (55.8)	34 (54.8)	25 (44.6)
Diabetes, n (%)	10 (21.3)	15(34.9)	15 (24.2)	17 (30.4)
Tobacco, n (%)	23 (48.9)	23 (53.5)	30 (48.4)	29 (51.8)
TC (mmol/L)	3.98±0.78	3.85±0.92	4.65±1.01*	4.31±0.82
TG (mmol/L)	1.46±0.74	1.96±0.99	2.17±1.47*	1.35±0.65
LDL-C (mmol/L)	2.13±0.72	2.04±0.79	2.58±0.91*	2.52±0.65
HDL-C (mmol/L)	1.17±0.27	1.14±0.25	1.12±0.40	1.10±0.41
GLU (mmol/L)	5.13±0.79	5.27±0.98	5.93±1.85*	6.44±1.84*
Gensini score	0	26.09±25.23*	27.65±23.44*	56.25±31.77*
LVEF (%)	64.49±3.81	57.12±10.67*	61.13±9.35	44.66±8.75*
CRP (mg/L)	2.1±1.2	3.9±1.4*	4.6±2.0*	5.5±2.1*
Medications, n (%)				
β-blockers	7 (14.9)	29 (67.4)*	36 (58.1)*	18 (32.1)
ACEI/ARB	12 (25.5)	19 (44.2)	22 (35.5)	23 (41.1)
CCB	19 (40.4)	21 (48.8)	40 (64.5)	25 (44.6)
Nitrates	19 (40.4)	36 (83.7)*	53 (85.5)*	30 (53.6)
Statins	10 (21.3)	37 (86.0)*	42 (67.7)*	33 (58.9)*
Aspirin	13 (27.7)	40 (93.0)*	60 (96.8)*	52 (92.9)*

The data are given as the mean±SD or number of patients. CPS: chest pain syndrome; SAP: stable angina; UAP: unstable angina; AMI: acute myocardial infarction; TC: total cholesterol; TG: total triglycerides; LDL-C: low-density lipoprotein cholesterol; HDL-C: high-density lipoprotein cholesterol; GLU: fasting glucose; LVEF: left ventricular ejection fraction; CRP: C-reactive protein; ACEI: angiotensin-converting enzyme inhibitor; ARB: angiotensin receptor blocker; CCB: Calcium channel blocker.

* P<0.05 vs. CPS.

### 2. Plasma Cytokines Concentrations in CAD Patients

**Figure 1:**
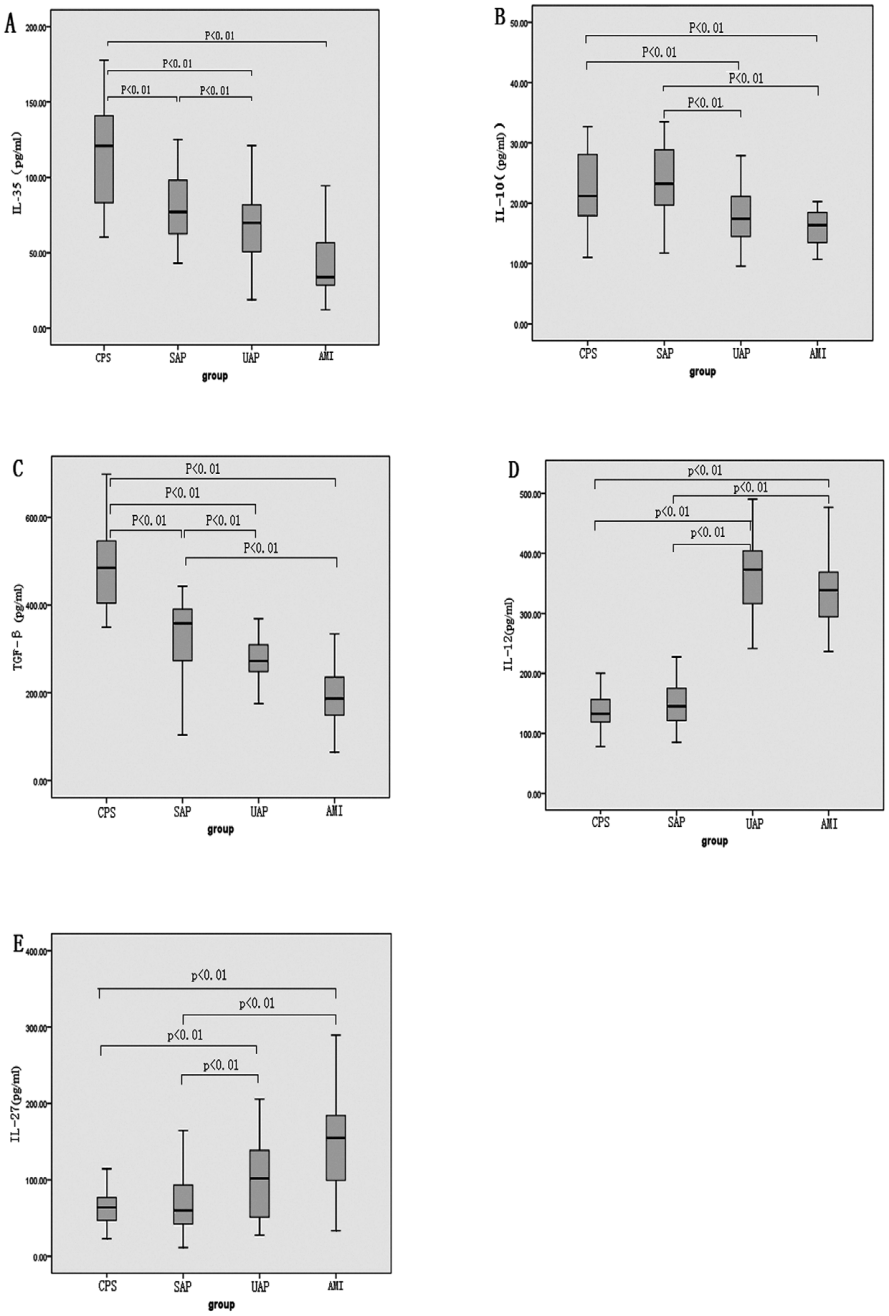
Cytokine concentrations in patients with CAD. A: The IL-35 concentrations in patients with AMI, UAP, and SAP were significantly decreased compared with those in patients with CPS. B: The IL-10 concentrations in patients with AMI and UAP were significantly decreased compared with those in patients with SAP and CPS. C: The TGF-β1 concentrations in patients with AMI, UAP, and SAP were significantly decreased compared with those in patients with CPS. D: The IL-12 concentrations in patients with AMI and UAP were significantly increased compared with those in patients with SAP and CPS. E: The IL-27 concentrations in patients with AMI and UAP were significantly increased compared with those in patients with SAP and CPS.

As shown in Table 2 and Figure 1, plasma IL-35, IL-10, TGF-β1, IL-12 and IL-27 levels were detected in each group. The IL-35 concentrations in patients with AMI, UAP, and SAP were significantly decreased compared with those in patients with chest pain syndrome. The IL-10 concentrations in patients with AMI and UAP were significantly lower than those in patients with SAP and chest pain syndrome. The TGF-β1 concentrations in patients with AMI, UAP and SAP were significantly lower than those in patients with chest pain syndrome. IL-12 and IL-27 levels in patients with AMI and UAP were significantly higher than those in patients with SAP and chest pain syndrome. To exclude potential inflammation from the chest pain syndrome group, 30 healthy college students (aged from 21 to 25 years) from Guangxi Medical University were also studied. There were no differences between the chest pain syndrome group and the healthy students (IL-35∶122.23±35.76 pg/ml, IL-10∶23.98±4.16 pg/ml, TGF-β1∶471.15±82.32 pg/ml, IL-12∶135.28±29.61 pg/ml, IL-27∶65.89±27.47 pg/ml). Furthermore, 208 patients were divided into a hypertensive group (111 cases) and a normotensive group (97 cases). The results showed that there was no significant difference in plasma IL-35 levels between the hypertensive group (76.06±34.28 pg/ml) and the normotensive group (84.07±40.49 pg/ml). In addition, there was no significant difference in plasma IL-35 levels between the diabetic group (57 cases, 81.87±41.32 pg/ml) and the non-diabetic group (151 cases, 79.02±35.97 pg/ml). Similar results were obtained for the other cytokines (data not shown).

**Table 2:** Plasma cytokines levels in each group.

	CPS	SAP	UAP	AMI
	(n = 47)	(n = 43)	(n = 62)	(n = 56)
IL-35 (pg/ml)	115.06±32.27	90.74±34.22**	72.20±26.63** ^,^ ##	50.21±24.69** ^,^ ##
IL-10 (pg/ml)	22.67±7.99	24.28±7.79	18.41±4.83** ^,^ ##	17.45±6.53** ^,^ ##
TGF-β1 (pg/ml)	490.99±96.39	339.44±77.68**	276.83±77.70** ^,^ ##	220.39±104.04** ^,^ ##
IL-12 (pg/ml)	138.68±34.37	153.84±53.86	349.72±85.22** ^,^ ##	318.05±86.82** ^,^ ##
IL-27 (pg/ml)	63.60±22.75	70.84±38.77	101.75±51.42** ^,^ ##	148.88±68.45** ^,^ ##

Note: The data are given as the mean±SD.

** P<0.01 vs. CPS,

# P<0.05 vs. SAP,

## P<0.01 vs. SAP.

### 3. Plasma Cytokines Concentration Effected by Aspirin and Clopidogrel

**Figure 2:**
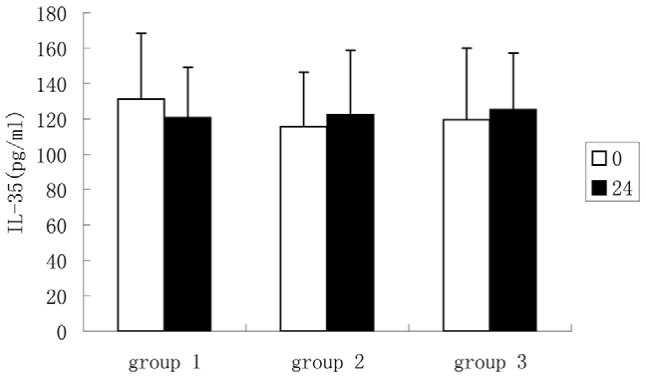
Plasma IL-35 concentration effected by aspirin and clopidogrel. Group 1 was treated with aspirin 100 mg once, Group 2 was treated with clopidogrel 75 mg once, and Group 3 was treated with aspirin 100 mg plus clopidogrel 75 mg once. IL-35 concentrations at before treatment (0) and 24 hours after treatment (24) were measured using an ELISA. The plasma IL-35 levels did not significantly change after treatment.

As shown in Figure 2, the plasma IL-35 levels did not significantly change after treatment with aspirin and clopidogrel. In addition, there was also no significant difference in plasma levels of IL-10, TGF-β1, IL-12 and IL-27 (data not shown).

### 4. Plasma Cytokines Concentration and other Measured Parameters

**Figure 3:**
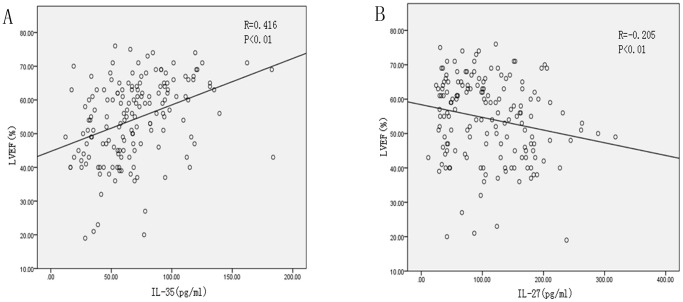
Spearman’s correlation between IL-35 and IL-27 concentrations and LVEF. A: IL-35 concentrations were positively correlated with LVEF in CAD (R = 0.416, P<0.01). B: IL-27 concentrations were negatively correlated with LVEF in CAD (R = −0.205, P<0.01).

We assessed whether the plasma cytokine levels were associated with the Gensini score used to quantify the severity of coronary artery stenosis in CAD. There was no significant correlation between the plasma cytokine levels and the Gensini score (data not shown). We further assessed whether plasma cytokine levels were associated with lipid and lipoprotein fractions (triglycerides, high-density lipoprotein cholesterol and low-density lipoprotein cholesterol), fasting glucose, CRP and LVEF in patients with CAD. The results showed that lower IL-35 levels were moderately positively correlated with LVEF in CAD patients (R = 0.416, *P*<0.01) whereas higher IL-27 levels were weakly negatively correlated with LVEF in CAD patients(R = −0.205, *P*<0.01) (Figure 3) but not with other parameters (data not shown).There was no significant correlation between the levels of other cytokines and other parameters mentioned above (data not shown).

## Discussion

In this study, the plasma levels of the novel anti-inflammatory cytokine IL-35, IL-10, TGF-β1, and two other IL-12 family members, IL-12 and IL-27, were investigated in CAD patients. The results showed that the levels of plasma IL-35, IL-10 and TGF-β1 were dramatically decreased, whereas plasma IL-12 and IL-27 levels were significantly increased in patients with UAP and AMI compared with chest pain syndrome patients. Some studies found that aspirin could efficiently regulate the secretion of cytokines *in vitro*, whereas some showed contrary results [27]–[29]. In this study, we did not find a significant change in plasma levels of IL-35 and other cytokines after treatment with aspirin and clopidogrel, suggesting that a longer time is required to observe the effect of aspirin and clopidogrel on IL-35 production *in vivo*. The relationship between the levels of each cytokine and the severity of coronary arteray stenosis was measured by Spearman’s correlation. We found that the levels of each cytokine were not related to the Gensini score, suggesting that changes in cytokine levels are associated with the inflammatory status and plaque destabilization of CAD but not the severity of the coronary artery stenosis. Previous studies have confirmed that coronary lesions in SAP patients are often characterized by severe luminal narrowing, mild inflammatory response and a stable plaque, while coronary lesions in acute coronary syndromes are often characterized by moderate luminal narrowing, a strong inflammatory response and a vulnerable plaque. In addition, lower IL-35 concentrations showed a positive correlation and higher IL-27 concentrations showed a negative correlation with LVEF in patients with CAD, suggesting a potential role of IL-35 and IL-27 in the prognosis of CAD.

Evidence from atherosclerosis-prone models shows that the development and progression of atherosclerosis is related to the imbalance of anti-inflammatory and pro-inflammatory cytokines [2], [3]. Pro-inflammatory cytokines, including IFN-γ and TNF-α, are mainly Th1-type cytokines and can be found even in the early stage of the lesion. These cytokines are significantly increased in the shoulder of vulnerable plaques, and constitute a large network system with inflammatory cells that amplify the inflammatory response, leading to plaque rupture and thrombosis. IL-12 is abundant in atherosclerotic plaques and is the principal cytokine that promotes the development of Th1 cells, which secrete IFN-γ and play critical pathogenic role in atherosclerosis [30]. The treatment of ApoE knockout mice with recombinant IL-12 aggravated atherosclerosis progression, and higher plasma IL-12 levels were also confirmed as a biomarker in CAD patients [4], [31]. Similar to IL-12, IL-27 positively induces Th1-type responses and negatively regulates Th2-type responses [32], [33]. Though the exact role of IL-27 in atherosclerosis is unknown, it is reasonable to hypothesize that IL-27 may participate in atherosclerosis and that higher plasma IL-27 levels could be the new biomarker of CAD [34]. However, the changes in serum anti-inflammatory cytokine levels are still controversial. Some studies suggest that the levels of serum anti-inflammatory cytokines were increased in ACS patients, while some studies observed that the levels of serum anti-inflammatory cytokines were significantly decreased [5]–[11], [35], [36]. These studies indicate that the levels of serum anti-inflammatory cytokines or the ratio of anti-inflammatory to pro-inflammatory cytokines can be used to predict the incidence of cardiovascular events and the prognosis of ACS patients. Evidence from the CAPTURE study showed that elevated serum IL-10 levels not only at baseline but also before discharge are associated with a favorable outcome in ACS patients [9]. In our previous study, we observed that plasma TGF-β1 levels in ACS patients were lower than those in control and SAP patients, while there was no notable difference in plasma TGF-β1 levels between chest pain syndrome patients and SAP patients [11]. A possible reason for this difference is that the SAP patients in the previous study were mostly Canadian Cardiovascular Society (CCS) class I–II, but the SAP patients treated with drug therapy were considered CCS class III–IV in this study. Therefore, the follow-up of these patients may be very meaningful. The data from this study demonstrate for the first time that the levels of plasma IL-35, a novel anti-inflammatory cytokine, are positively correlated with LVEF in patients with CAD, which is an independent predictor of recurrent coronary events and prognosis in CAD patients. Therefore, decreased plasma IL-35 levels not only represent insufficient anti-inflammatory activity *in vivo* but also hold promise as a novel biomarker to assess the prognosis of CAD patients.

IL-35 is an IL-12 family member cytokine composed of an α chain p35 and a β chain EBI3 [12], [13].The two subunits also form components of other cytokines. The p35 subunit binds with a p40 subunit to form IL-12 while the EBI3 sub-unit binds with a p28 subunit to form IL-27, another member of the IL-12 family. EBI3 induced in B-lymphocytes by EBV infection encodes a 34-kDa glycoprotein homologous to the p40 subunit of IL-12. EBI3 is expressed at high levels in placental trophoblast cells, activated dendritic cells and lymphocytes and at lower levels in macrophages and endothelial cells, but not in normal resting CD3+ T cells in humans [14], [20]. On the other hand, the p35 gene is constitutively expressed at low levels in many cell types. It has been found that a large number of p35 subunits are co-expressed with EBI3. Both *Ebi3* and *p35* knockout mice show overt autoimmunity or inflammatory disease, suggesting that the EBI3/p35 heterodimer may be an important immunomodulator [14], [15], [37]. The EBI3/p35 heterodimer, which is currently designated as IL-35, has been confirmed to suppress Teff cell activity, expand the effect of Treg cells and attenuate established collagen-induced arthritis [12]. Collision et al. found that both EBI3 and p35 are highly expressed and constitutively secreted by mouse Foxp3+ Treg cells but not by activated Teff cells [13]. Furthermore, the regulatory activity of Treg cells from *Ebi3* or *p35* knockout mice was significantly reduced compared to that of wild-type Treg cells *in vivo* and *in vitro,* suggesting that IL-35 is critical for the regulatory activity of Treg cells [13], [38], [39]. The role of IL-35 in human Treg cells, however, is more complicated. Studies performed by Devergne et al. and Allan et al. showed that IL-35 is not constitutively expressed by human Treg cells but is instead expressed by activated Teff cells and macrophages, indicating that IL-35 may not be related to the suppressive mechanism of human Treg [20], [21]. However, when stimulated by anti-CD3 and anti-CD28, the expression of both EBI3 and p35 in human Treg cells was significantly higher than that in Teff cells. A neutralizing anti-IL-35 antibody completely abolished the suppression of human Treg cells, suggesting that the difference in the role of IL-35 in human Treg cells observed by these studies may be due to the timing of the analysis, the purification techniques, and/or the stimulation used [22]. Furthermore, IL-35 was shown to efficiently induce the conversion of suppressed target Teff cells into the Foxp3-independent Treg population, namely iTr35, in both human and mice [22], [23], [40], [41]. When co-cultured with dendritic cells activated by human rhinovirus (R-DC), iTr35 can also be induced to secrete IL-35. This effect could be reversed by blocking of inhibitory receptors B7-H1 and sialoadhesin on R-DC, suggesting an important mechanism in regulating the IL-35 expression [40]. In addition to inducing the generation of iTr35 cells and suppressing the proliferation of Teff cells, IL-35 performs its biological effect via up-regulating the expression of anti-inflammatory cytokines such as IL-10 and IL-35 and directly inhibiting the activity of other target cells.

Recent studies on IL-35 have focused on disease-prone models and healthy populations, but the role of IL-35 in coronary artery disease has yet to be understood. Liu et al. found that EBI3 and p35 are highly expressed in CD4+T cells from chronic hepatitis B patients, which may contribute to the immune escape of HBV [42]. However, they did not measure the plasma IL-35 levels in their patients. It has been shown that EBV-specific T lymphocytes can be frequently observed in human atherosclerotic plaques, suggesting the ability of these T lymphocytes to secrete IL-35 or IL-27 [43]. In fact, the expression of p35 was only found in the brain, intestine, and spleen, while the expression of EBI3 was found in the placenta, eye, lymph node, and pancreas; however, neither subunit was expressed in the heart or vessels of healthy human subjects [44]. However, IL-35 could be up-regulated following induction in human tissue [26]. A recent study revealed that EBI3 and p35 are expressed in almost all advanced plaque lesions and are co-expressed in atheroma vascular smooth muscle cell, indicating that IL-35 may be secreted by vascular smooth muscle cells [26]. Stimulated by the pro-inflammatory cytokines TNF-α or IFN-γ or both, the expression of EBI3 and p35 increased and this effect was attenuated by pretreatment with peroxisome proliferator-activated receptor-γ (PPARγ) agonist rosiglitazone, suggesting a potential role of IL-35 in the progression of atherosclerosis. When we considered this observation together with our research, it raised a new question: why is the expression of IL-35 up-regulated in plaques but down-regulated in peripheral blood? We speculate that plasma IL-35 is mostly secreted by Treg cells and that the suppressive function of peripheral Treg cells is significantly reduced in acute coronary syndromes [45].

To the best of our knowledge, our study is the first to measure the plasma IL-35 levels in CAD. In conclusion, the results of this study show that the levels of plasma IL-35 are dramatically decreased and positively correlated with LVEF in patients with CAD. Our study also creates two new areas of investigation, namely, the potential of IL-35 as a novel biomarker to assess the onset and prognosis of CAD, and second, IL-35 gene regulation as a highly effective therapeutic tool for the treatment of atherosclerosis and CAD. In prospective studies, we will recruit more patients to observe the changes of plasma IL-35 and confirm the relationship between these changes and the prognosis of CAD. However, the exact role of IL-35 in the atherosclerosis process and in the onset of CAD has yet to be elucidated. Further studies are required to investigate the precise effect and the signaling transduction mechanisms of IL-35 in the atherosclerosis process.

## References

[1] Ross R (1999) Atherosclerosis–an inflammatory disease. N Engl J Med 340: 115–126. 9887164 10.1056/NEJM199901143400207

[2] Frostegård J, Ulfgren AK, Nyberg P, Hedin U, Swedenborg J, et al (1999) Cytokine expression in advanced human atherosclerotic plaques: dominance of pro-inflammatory (Th1) and macrophage-stimulating cytokines. Atherosclerosis 145: 33–43. 10428293 10.1016/s0021-9150(99)00011-8

[3] Tedgui A, Mallat Z (2006) Cytokines in Atherosclerosis: Pathogenic and Regulatory Pathways. Physiol Rev 86: 515–581. 16601268 10.1152/physrev.00024.2005

[4] Alam SE, Nasser SS, Fernainy KE, Habib AA, Badr KF (2004) Cytokine imbalance in acute coronary syndrome. Curr Opin Pharmacol 4: 166–170. 15063361 10.1016/j.coph.2003.10.011

[5] Smith DA, Irving SD, Sheldon J, Cole D, Kaski JC (2001) Serum levels of the antiinflammatory cytokine interleukin-10 are decreased in patients with unstable angina. Circulation 104: 746–749. 11502695 10.1161/hc3201.094973

[6] Mazzone A, De Servi S, Vezzoli M, Fossati G, Mazzucchelli I, et al (1999) Plasma levels of interleukin 2, 6, 10 and phenotypic characterization of circulating T lymphocytes in ischemic heart disease. Atherosclerosis 145: 369–374. 10488965 10.1016/s0021-9150(99)00104-5

[7] Anguera I, Miranda-Guardiola F, Bosch X, Filella X, Sitges M, et al (2002) Elevation of serum levels of the anti-inflammatory cytokine interleukin-10 and decreased risk of coronary events in patients with unstable angina. Am Heart J 144: 811–817. 12422149 10.1067/mhj.2002.124831

[8] Kilic T, Ural D, Ural E, Yumuk Z, Agacdiken A, et al (2006) Relation between proinflammatory to anti-inflammatory cytokine ratios and long-term prognosis in patients with non-ST elevation acute coronary syndrome. Heart 92: 1041–1046. 16547209 10.1136/hrt.2005.080382 PMC1861097

[9] Heeschen C, Dimmeler S, Hamm CW, Fichtlscherer S, Boersma E, et al (2003) Serum level of the antiinflammatory cytokine interleukin-10 is an important prognostic determinant in patients with acute coronary syndromes. Circulation 107: 2109–2114. 12668510 10.1161/01.CIR.0000065232.57371.25

[10] Lebastchi AH, Qin L, Khan SF, Zhou J, Geirsson A, et al (2011) Activation of human vascular cells decreases their expression of transforming growth factor-beta. Atherosclerosis 219: 417–424. 21862019 10.1016/j.atherosclerosis.2011.07.121 PMC3226933

[11] Ji QW, Guo M, Zheng JS, Mao XB, Peng YD, et al (2009) Downregulation of T helper cell type 3 in patients with acute coronary syndrome. Arch Med Res 40: 285–293. 19608018 10.1016/j.arcmed.2009.04.002

[12] Niedbala W, Wei XQ, Cai B, Hueber AJ, Leung BP, et al (2007) IL-35 is a novel cytokine with therapeutic effects against collagen-induced arthritis through the expansion of regulatory T cells and suppression of Th17 cells. Eur J Immunol 37: 3021–3029. 17874423 10.1002/eji.200737810

[13] Collison LW, Workman CJ, Kuo TT, Boyd K, Wang Y, et al (2007) The inhibitory cytokine IL-35 contributes to regulatory T-cell function. Nature 450: 566–569. 18033300 10.1038/nature06306

[14] Devergne O, Hummel M, Koeppen H, Le Beau MM, Nathanson EC, et al (1996) A novel interleukin-12 p40-related protein induced by latent Epstein-Barr virus infection in B lymphocytes. J Virol 70: 1143–1145. 8551575 10.1128/jvi.70.2.1143-1153.1996 PMC189923

[15] Devergne O, Birkenbach M, Kieff E (1997) Epstein-Barr virus-induced gene 3 and the p35 subunit of interleukin 12 form a novel heterodimeric hematopoietin. Proc Natl Acad Sci U S A 94: 12041–12046. 9342359 10.1073/pnas.94.22.12041 PMC23696

[16] Collison LW, Pillai MR, Chaturvedi V, Vignali DA (2009) Regulatory T cell suppression is potentiated by target T cells in a cell contact, IL-35- and IL-10-dependent manner. J Immunol 182: 6121–6128. 19414764 10.4049/jimmunol.0803646 PMC2698997

[17] Huang CH, Loo EX, Kuo IC, Soh GH, Goh DL, et al (2011) Airway inflammation and IgE production induced by dust mite allergen-specific memory/effector Th2 cell line can be effectively attenuated by IL-35. J Immunol 187: 462–471. 21613618 10.4049/jimmunol.1100259

[18] Wirtz S, Billmeier U, Mchedlidze T, Blumberg RS, Neurath MF (2011) Interleukin-35 mediates mucosal immune responses that protect against T-cell-dependent colitis. Gastroenterology 141: 1875–1886. 21820391 10.1053/j.gastro.2011.07.040 PMC3624892

[19] Whitehead GS, Wilson RH, Nakano K, Burch LH, Nakano H, et al (2012) IL-35 production by inducible costimulator (ICOS)-positive regulatory T cells reverses established IL-17-dependent allergic airways disease. J Allergy Clin Immunol 129: 207–215. 21906794 10.1016/j.jaci.2011.08.009 PMC3269135

[20] Bardel E, Larousserie F, Charlot-Rabiega P, Coulomb-L’Herminé A, Devergne O (2008) Human CD4+ CD25+ Foxp3+ regulatory T cells do not constitutively express IL-35. J Immunol 181: 6898–6905. 18981109 10.4049/jimmunol.181.10.6898

[21] Allan SE, Song-Zhao GX, Abraham T, McMurchy AN, Levings MK (2008) Inducible reprogramming of human T cells into Treg cells by a conditionally active form of FOXP3. Eur J Immunol 38: 3282–3289. 19039775 10.1002/eji.200838373

[22] Chaturvedi V, Collison LW, Guy CS, Workman CJ, Vignali DA (2011) Cutting edge: Human regulatory T cells require IL-35 to mediate suppression and infectious tolerance. J Immunol 186: 6661–6666. 21576509 10.4049/jimmunol.1100315 PMC3110563

[23] Collison LW, Chaturvedi V, Henderson AL, Giacomin PR, Guy C, et al (2010) IL-35-mediated induction of a potent regulatory T cell population. Nat Immunol 11: 1093–1101. 20953201 10.1038/ni.1952 PMC3008395

[24] Kochetkova I, Golden S, Holderness K, Callis G, Pascual DW (2010) IL-35 stimulation of CD39+ regulatory T cells confers protection against collagen II-induced arthritis via the production of IL-10. J Immunol 184: 7144–7153. 20483737 10.4049/jimmunol.0902739 PMC3145775

[25] Bettini M, Castellaw AH, Lennon GP, Burton AR, Vignali DA (2012) Prevention of Autoimmune Diabetes by Ectopic Pancreatic β-Cell Expression of Interleukin-35. Diabetes. [Epub ahead of print]. 10.2337/db11-0784 PMC3357277 22427377

[26] Kempe S, Heinz P, Kokai E, Devergne O, Marx N, et al (2009) Epstein-barr virus-induced gene-3 is expressed in human atheroma plaques. Am J Pathol 175: 440–447. 19556516 10.2353/ajpath.2009.080752 PMC2708829

[27] Redondo S, Ruiz E, Gordillo-Moscoso A, Navarro-Dorado J, Ramajo M, et al (2010) Role of TGF-β1 and MAP Kinases in the Antiproliferative Effect of Aspirin in Human Vascular Smooth Muscle Cells. PLoS ONE 5: e9800. 20339548 10.1371/journal.pone.0009800 PMC2842433

[28] Block RC, Dier U, Calderonartero P, Shearer GC, Kakinami L, et al (2012) The effects of EPA+ DHA and aspirin on inflammatory cytokines and angiogenesis factors. World J Cardiovasc Dis 2: 14–19. 22530200 10.4236/wjcd.2012.21003 PMC3331709

[29] Hovens MM, Snoep JD, Groeneveld Y, Frölich M, Tamsma JT, et al (2008) Effects of aspirin on serum C-reactive protein and interkin-6 levels in patients with type 2 diabetes without cardiovascular disease : a randomized placebo-controlled cross over trial. Diabetes Obes Metab 10: 668–674. 18034847 10.1111/j.1463-1326.2007.00794.x

[30] Uyemura K, Demer LL, Castle SC, Jullien D, Berliner JA, et al (1996) Cross-regulatory roles of interleukin (IL)-12 and IL-10 in atherosclerosis. J Clin Invest 97: 2130–8. 8621803 10.1172/JCI118650 PMC507288

[31] Lee TS, Yen HC, Pan CC, Chau LY (1999) The role of interleukin 12 in the development of atherosclerosis in ApoE-deficient mice. Arterioscler Thromb Vasc Biol 19: 734–742. 10073981 10.1161/01.atv.19.3.734

[32] Cao Y, Doodes PD, Glant TT, Finnegan A (2008) IL-27 induces a Th1 immune response and susceptibility to experimental arthritis. J Immunol 180: 922–30. 18178832 10.4049/jimmunol.180.2.922

[33] Tassi E, Braga M, Longhi R, Gavazzi F, Parmiani G, et al (2009) Non-redundant role for IL-12 and IL-27 in modulating Th2 polarization of carcinoembryonic antigen specific CD4 T cells from pancreatic cancer patients. PLoS One 4: e7234. 19798410 10.1371/journal.pone.0007234 PMC2749205

[34] Jafarzadeh A, Nemati M, Rezayati MT (2011) Serum levels of interleukin (IL)-27 in patients with ischemic heart disease. Cytokine 56: 153–6. 21795063 10.1016/j.cyto.2011.06.014

[35] Tziakas DN, Chalikias GK, Hatzinikolaou HI, Parissis JT, Papadopoulos ED, et al (2003) Anti-inflammatory cytokine profile in acute coronary syndromes: behavior of interleukin-10 in association with serum metalloproteinases and proinflammatory cytokines. Int J Cardiol 92: 169–175. 14659849 10.1016/s0167-5273(03)00084-6

[36] Bogavac-Stanojevic N, Djurovic S, Jelic-Ivanovic Z, Spasojevic-Kalimanovska V, Kalimanovska-Ostric D (2003) Circulating transforming growth factor-beta1, lipoprotein(a) and cellular adhesion molecules in angiographically assessed coronary artery disease. Clin Chem Lab Med 41: 893–898. 12940514 10.1515/CCLM.2003.135

[37] Niedobitek G, Päzolt D, Teichmann M, Devergne O (2002) Frequent expression of the Epstein-Barr virus (EBV)-induced gene, EBI3, an IL-12 p40-related cytokine, in Hodgkin and Reed-Sternberg cells. J Pathol 198: 310–316. 12375263 10.1002/path.1217

[38] Yang J, Yang M, Htut TM, Ouyang X, Hanidu A, et al (2008) Epstein-Barr virus-induced gene 3 negatively regulates IL-17, IL-22 and RORgamma t. Eur J Immunol 38: 1204–1214. 18412165 10.1002/eji.200838145 PMC2989250

[39] Liu JQ, Liu Z, Zhang X, Shi Y, Talebian F, et al (2012) Increased Th17 and regulatory T cell responses in EBV-induced gene 3-deficient mice lead to marginally enhanced development of autoimmune encephalomyelitis. J Immunol 188: 3099–3106. 22387555 10.4049/jimmunol.1100106 PMC3311737

[40] Seyerl M, Kirchberger S, Majdic O, Seipelt J, Jindra C, et al (2010) Human rhinoviruses induce IL-35-producing Treg via induction of B7-H1 (CD274) and sialoadhesin (CD169) on DC. Eur J Immunol 40: 321–329. 19950173 10.1002/eji.200939527

[41] Collison LW, Delgoffe GM, Guy CS, Vignali KM, Chaturvedi V, et al (2012) The composition and signaling of the IL-35 receptor are unconventional. Nat Immunol 13: 290–299. 22306691 10.1038/ni.2227 PMC3529151

[42] Liu F, Tong F, He Y, Liu H (2011) Detectable expression of IL-35 in CD4+ T cells from peripheral blood of chronic hepatitis B patients. Clin Immunol 139: 1–5. 21285006 10.1016/j.clim.2010.12.012

[43] de Boer OJ, Teeling P, Idu MM, Becker AE, van der Wal AC (2006) Epstein Barr virus specific T-cells generated from unstable human atherosclerotic lesions: implications for plaque inflammation. Atherosclerosis 184: 322–329. 15941569 10.1016/j.atherosclerosis.2005.05.001

[44] Li X, Mai J, Virtue A, Yin Y, Gong R, et al (2012) IL-35 is a novel responsive anti-inflammatory cytokine–a new system of categorizing anti-inflammatory cytokines. PLoS One 7: e33628. 22438968 10.1371/journal.pone.0033628 PMC3306427

[45] Mor A, Luboshits G, Planer D, Keren G, George J (2006) Altered status of CD4(+)CD25(+) regulatory T cells in patients with acute coronary syndromes. Eur Heart J 27: 2530–2537. 16954132 10.1093/eurheartj/ehl222

